# Factors Associated With Pathogenicity of Anti-Glomerular Basal Membrane Antibodies: A Case Report

**DOI:** 10.1097/MD.0000000000003654

**Published:** 2016-05-13

**Authors:** Rime Ossman, David Buob, Thomas Hellmark, Isabelle Brocheriou, Julie Peltier, Ryad Tamouza, Karine Dahan, Alexandre Hertig, Eric Rondeau, Pierre Galichon

**Affiliations:** From the Urgences Néphrologiques et Transplantation Rénale (RO, JP, AH, ER, PG); Service d’Anatomie Pathologique (DB, IB); Service de Néphrologie et Dialyses (KD), Hôpital Tenon; Université Pierre et Marie Curie (DB, IB, AH, ER, PG), (Paris 6), Sorbonne Universités, Paris, France; Department of Nephrology (TH), Clinical Sciences in Lund, Lund University, Lund, Sweden; and Laboratoire Jean Dausset (LabEX Transplantex) et Inserm UMRS 1160 (RT), Hôpital Saint Louis, Paris, France.

## Abstract

Antiglomerular basement membrane (GBM) disease is known as a super-acute proliferative glomerulonephritis caused by auto-antibodies targeting the NC1 domain of the α3 chain of type IV collagen.

Here, we describe a case of atypical anti-GBM disease presenting as a dialysis-dependent acute renal failure with unusual mild glomerular involvement. We found that immunoglobulin G (IgG) deposits were restricted to the uncommon IgG2 and IgG4 subclasses, and that blood was positive for anti-GBM antibodies by immunofluorescence, but not by Enzyme Linked Immunosorbent Assay (ELISA). The patient was treated with plasma exchanges, corticosteroids, and cyclosphosphamide. He eventually regained a normal renal function.

This case demonstrates that biopsy-proven anti-GBM disease can have reduced pathogenicity. Referring to previous studies of anti-GBM detection in the blood from healthy or minimally ill individuals, we discuss the antigenic specificities, the IgG subclasses, and the involvement of complement in this observation.

We suggest that anti-GBM disease is a heterogeneous entity and that the study of IgG subclasses by immunofluorescence may help to distinguish categories with different severities.

## Abstract 2

Supplemental Digital Content is available in the text

## INTRODUCTION

Antiglomerular basement membrane (GBM) disease involves the kidney (glomerulonephritis) and the lung (alveolitis). It is associated with anti-GBM antibodies targeting the same antigen in the GBM and alveolar basal membrane: the noncollagenous domain of the α3 chain of type IV collagen (NC1). Anti-GBM disease is usually characterized by severe necrotizing glomerulonephritis, with detection by immunostaining of the antibody as a linear deposition of immunoglobulin G (IgG) along the GBM. In addition to immunostaining in renal biopsy specimens, anti-GBM antibodies can be detected in blood either by immunofluorescence after incubation on primate kidney slides or by an ELISA test specific to NC1 monomers. Anti-GBM disease typically results in severe kidney injury that often progresses to end-stage renal disease despite aggressive immunosuppressive therapy and plasmapheresis.^1^ The anti-GBM antibodies are instrumental in the pathophysiology of anti-GBM disease, with a putative role of complement activation suggested by the frequent presence of complement deposits within the glomeruli and increased C5b9 urinary excretion. In the typical necrotizing glomerulonephritis, anti-GBM IgGs were reported to be predominantly IgG1 or IgG3.^2–4^ An atypical presentation with surprisingly mild glomerular involvement in patients with biopsy-proven linear GBM IgG4 antibodies has been recently described.^5^ We report here a comprehensive case of anti-GBM disease with a unique pattern of severe renal failure and mild pathological involvement, typical anti-GBM IgG immunostaining, but with typical IgG subclasses and atypical serum anti-GBM tests. Collectively, these features suggest that complement activation by anti-GBM antibodies may contribute to the severity of this disease.

## METHODS

### Patient Information

A 48-year-old man consulted the emergency department for acute renal failure. He reported a medical history of hypertension, heavy smoking, obesity with body mass index 40 kg/m^2^, sleep apnea, multiple venous thromboses, and a pulmonary embolism 7 years ago treated by a vitamin K antagonist (fluindione). He had normal creatininemia values until 2 months before referral.

### Clinical Findings

Eight days previously, he had been admitted to the emergency room of another hospital with progressive symptoms of fatigue, gross hematuria, coughing, and hemoptysis, 2 days after being treated for a dental abscess with amoxicillin/clavulanate and a nonsteroidal anti-inflammatory drug. He reported no history of infectious episode or exposition to toxic fumes. He was referred to our department for rapidly progressive glomerulonephritis.

### Timeline

**FIGURE 1:**
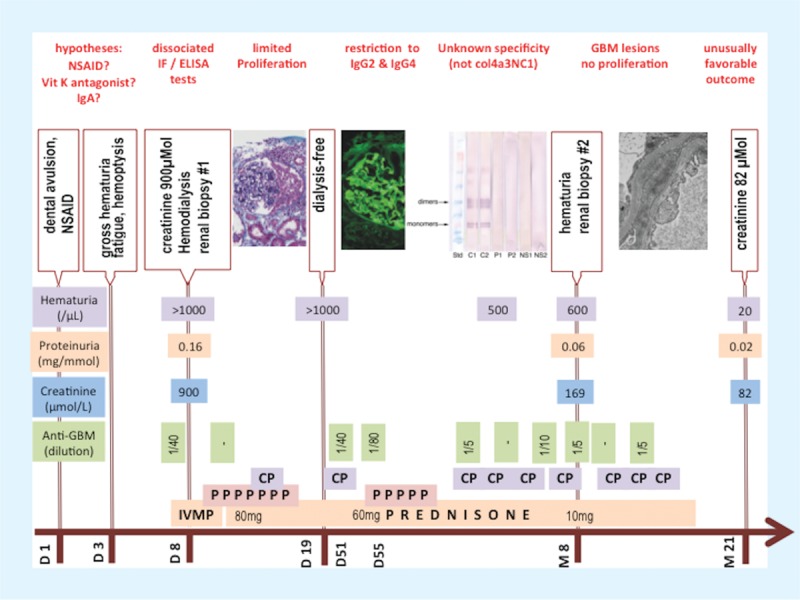
Timeline of the case. On the time axis, D stands for day and M for month. CP = cyclophosphamide, IVMP = intravenous methylprednisone, NSAID = nonsteroidal anti-inflammatory drug, PE = plasma exchange.

The timeline of this case is represented on Figure 1.

## RESULTS

### Diagnostic Assessment

**FIGURE 2:**
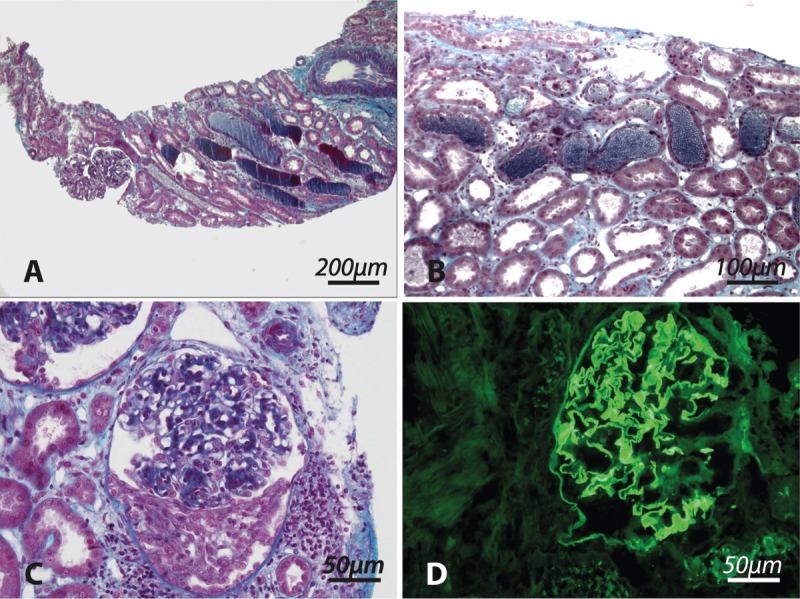
Biopsy No. 1. A–C, Masson trichrome staining. D, Immunoglobulin G (IgG) immunostaining. A, Intense acute tubular necrosis with permeable glomeruli and no fibrosis. B, Acute tubular necrosis with hemorrhagic tubules. C, The only glomerular anomaly observed on the biopsy. D, Diffuse linear immunostaining of the glomerular basal membrane for IgG.

**FIGURE 3:**
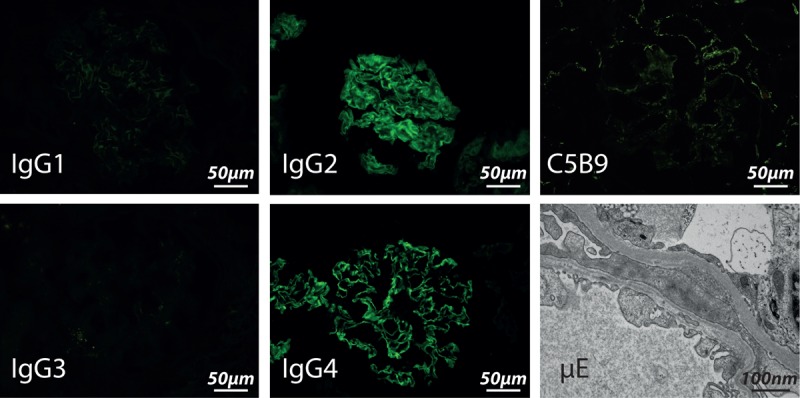
Biopsy No. 2. Immunostaining on glomeruli is absent for IgG1 and IgG3, and diffuse for IgG2 and IgG4. C5B9 immunofluorescence staining is negative. Electronic microscopy (μE) showing the irregular width of the glomerular basal membrane.

Laboratory investigations revealed acute kidney failure with a creatinine value of 900 μmol/L, and urinalysis was characteristic of glomerular disease with the presence of acanthocytes and non-nephrotic proteinuria (proteinuria/creatininuria 0.16 g/mmol). Titers for anti-GBM antibodies in blood were positive by immunofluorescence (titer 1/80), but were negative using ELISA, indicating a different specificity than the usual NC1 fragment of col4a3 (Supplemental material), whereas the remaining immunological tests were normal including complement (C3: 1.15 g/L, C4: 0.31 g/L) and negative Anti Neutrophil Cytoplasmic Antibody. The Human Leucocyte Antigen (HLA) class I and class II genotypes were as follows: HLA-A^∗^11/29, B^∗^07/51, C^∗^15/15, and HLA-DRB1^∗^04/04, DQB1^∗^03/03, respectively. He had a mild normocytic anemia (hemoglobin 11.8 g/dL), and the chest tomodensitometry showed no alveolar infiltrates. Examination of the renal biopsy specimen using light microscopy showed focal necrotizing glomerulonephritis, involving only one of the 12 glomeruli, in conjunction with tubular changes including acute tubular necrosis, and abundant red blood cell casts (Figure 2 A–C). There was no evidence of endocapillary proliferation or vascular damage, and there was no interstitial inflammation (except in exceptional fibrotic areas). The diagnosis of anti-GBM disease was confirmed by immunofluorescence which showed segmentation of the GBM with intense linear IgG deposition (Figure 2D). The study of IgG subclasses by immunofluorescence detected IgG2 and IgG4 on the GBM, but not IgG1 and IgG3. Immunofluorescence staining of C3 and C5b9 was negative in the glomeruli (Figure 3).

### Therapeutic Intervention

The patient was treated with hemodialysis, 3 intravenous steroid pulses (1000 mg), and 11 plasma exchanges (60 mL/kg), followed by oral steroid therapy (1 mg/kg/d) and 6 intravenous cyclophosphamide infusions (700 mg).

### Follow-up and Outcomes

Blood anti-GBM titers became negative and renal function partially recovered (creatininemia 169 μmol/L). Because of persisting symptoms of glomerulonephritis after 8 months (proteinuria/creatininuria 0.12 g/mmol and 35 acanthocytes/μL), the patient had a second renal biopsy. The biopsy specimen contained 11 glomeruli, which revealed no active lesions using light microscopy, but still the linear IgG deposition on the GBM by immunofluorescence. Intratubular red blood cells were still detectable. Electron microscopy revealed diffuse abnormalities of the GBM, which appeared irregularly-sized wit, marked segmental thinning (Figure 3). The study by immunofluorescence of alpha 1, 3, and 5 chains of collagen IV was normal.

Cyclophosphamide was continued up to a total of 9 pulses, and creatininemia recovered to normal values (82 μmol/L), as did proteinuria (0.16 g/d), 21 months after the occurrence of the disease.

## DISCUSSION

This case of anti-GBM disease is very unusual for several reasons: unusually slight necrotizing glomerulonephritis, because only 1 crescent (out of a total of 12 glomeruli) was detected after multiple sections of the first biopsy specimen; anti-GBM antibodies were detected in serum using immunofluorescence, but were negative by ELISA; immunofluorescence IgG subclass analysis on the renal biopsy specimen showed IgG4 and IgG2 restriction.

Pathophysiologically speaking, the IgG subclass specificity and the negativity of the ELISA test would seem logical. In 1987, Bowman et al^2^ reported that the clinical manifestations of the disease were associated with IgG1, but not IgG4 anti-GBM antibodies. More recently, the presence of anti-GBM antibodies in the serum of healthy blood donors was reported.^6^ The same team published the results from a cohort of anti-GBM disease patient subgroups divided according to renal function, and found that the severity of anti-GBM disease was associated with higher titers and a higher affinity to anti-GBM antibodies, and also to IgG1 and IgG3 subclasses.^7^ Complement activation has been suggested as an important mechanism in renal damage in anti-GBM disease,^8^ and the fact that IgG2 and IgG4 are weak complement activators supports this hypothesis and may explain the absence of severe glomerular injury in the case of our patient (the severity of the acute kidney injury may rather be linked to acute tubular necrosis and intratubular red blood cell casts caused by the use of nonsteroidal anti-inflammatory drugs and vitamin K antagonists). Perhaps a weaker immunosuppressive regimen may have yielded similar results. As IgG2 and IgG4 come after IgG1 and IgG3 in the sequence of isotypic switch, we expect that our patient will not progress towards a more severe presentation with complement activating anti-GBM. The reduced severity of anti-GBM disease when anti-GBM antibodies do not activate the complement system raises the question of minimization of immunosuppression in these cases and of complement-inhibiting therapies in anti-GBM disease with classical severe crescentic glomerulonephritis. Considering the lack of controlled clinical trials for such a rare disease and the poor renal prognosis of anti-GBM,^9^ we suggest that complement inhibition could be discussed on an individual basis for severe cases.

These “benign” IgG2 and IgG4 anti-GBM antibodies were found to react with a GBM antigen only in nondenaturing conditions, later identified as α345NC1 hexamers, and not NC1 monomers.^10,11^ This antigen specificity, present in primate kidney slides, but not on NC1 monomer-coated plates, will allow the detection of anti-GBM antibodies by immunofluorescence, but not by ELISA (there is no commercially available ELISA specific to α345NC1 hexamers). We found a similar disparity between immunofluorescence and ELISA in the case we describe here. The absence of the classically associated HLA DRB1^∗^15 allele further outlines the atypical character of this case. Indeed, both HLA DRB1^∗^04 and HLA-DQB1^∗^03 specificities (alone or in combination) are well known to be associated with several autoimmune disorders including diabetes or rheumatoid arthritis. Such nonspecific burden of autoimmunity may account for the different specificity of these antibodies.^12,13^

The ultrastructural abnormalities of the GBM found in the second renal biopsy specimen are another interesting finding of this case report. One could hypothesize that these alterations were caused by the fixation of the anti-GBM antibody. Notably, it should be reminded that similar ultrastructural anomalies of the GBM have been previously reported in anti-GBM disease.^14,15^ So, it is possible that the atypical anti-GBM antibodies are unable to cause severe glomerular necrosis on one hand, but, on the other hand, might gradually induce ultrastructural alterations of the GBM. These changes may explain the persisting hematuria in our patient in the absence of glomerular necrosis on the second biopsy, although the possibility that unsampled focal glomerulonephritis or the use of vitamin K antagonists may be the cause for the intratubular red blood cell casts should be kept in mind.

Thus, anti-GBM diseases encompass subgroups with different clinical, biological, and pathological features which, in turn, have significant prognostic implications. We suggest that IgG subclasses should be systematically assessed in patients with anti-GBM antibody glomerulonephritis.

### Informed Consent

The patient gave written informed consent to studying and reporting his case for scientific purposes.

### Care Statement

This case report follows the CAse REports guidelines for transparency and accuracy in the publication of case reports.^16^

## Supplementary Material

Supplemental Digital Content
