# Supplementary material for: Factors Associated With Pathogenicity of Anti-Glomerular Basal Membrane Antibodies: A Case Report
Source: Medicine (Baltimore). 2016 May 13;95(19):e3654. doi: 10.1097/MD.0000000000003654 (PMC4902534; doi:10.1097/MD.0000000000003654)
Supplement: Supplemental Digital Content [file medi-95-e3654-s001.doc]

### Immunostaining

Immunostainings were performed on 3µm-thick cryostat sections. Slides were incubated twice with PBS1X for 10 minutes followed by the FITC-conjugated primary antibody at 1/100 dilution (IgG: Dako #F0202, IgG1-4: Sigma-Aldrich #F0767 #F4516 #F4641 #F9890). Then we washed 3 times with PBS 1X for 10 minutes and mounted the slides with coverslips and Dako fluorescence mounting medium (#S3023).

### Enzyme linked immunosorbent assay (ELISA)

Polystyrene microtitre plates (NUNC Immunoplate, Roskilde, Denmark) were coated with 1.0 µg/well of purified type IV collagen from human kidneys, bovine testis or recombinant alpha 1 and alpha5(IV) NC1 domains in coating buffer (50mM sodium carbonate, pH 9.0) over night at 4°C. The plates were washed three times with washing buffer (0.15 mM NaCl, 0.05% (v/v) Tween 20) and then incubated for one hour in RT with 100 µl/well of human plasma diluted 1:50 in PBS-BSA (1.5 mM KH_2_PO_4_, 8 mM Na_2_HPO_4_, 0.12 M NaCl, 2.5 mM KCl, 0.05% (w/v) NaN_3_, containing 0.02% (w/v) bovine serum albumin, pH 7.3). After three new washes the plates were incubated for 1 h with 100 µl/well of alkaline phosphatase-conjugated goat anti-human IgG diluted 1/15 000 in PBS-BSA. The amount of bound antibodies was detected by the use of P-Nitrophenyl Phosphate (Sigma Chemical Company) (1 mg/ml) in substrate buffer (1 M Diethanolamine, 0.5 mM MgCl_2_, pH 9.8), as substrate. Colour development was measured spectrophotometrically at 405 nm.

### Western blotting

Purified human type IV collagen NC1 domains were electrophoresed on a 4-12% SDS polyacrylamide gel at 20 mA under non-reducing condition. Then, the proteins were transferred to a nitrocellulose paper (Schleicher & Schuell, Maidstone, Kent, UK) by an electrophoretic semi-dry blotting system (Pharmacia, Uppsala, Sweden) at 0.08mA/cm2 for 60 min. The nitrocellulose paper was blocked in PBS buffer with 1% BSA for 30 min at room temperature and cut into strips. The strips were incubated with the sera diluted 1:50, in PBS-BSA buffer at 4**°**C overnight. After three washes with TBST, the strips were incubated with alkaline phosphatase-conjugated secondary antibodies as described above, diluted 1:6,000 in PBS-BSA for 1 h at room temperature. In some experiments the secondary antibody was exchanged to a mouse anti-IgG4 monoclonal antibody followed by a rabbit-anti-mouse IgG-AP. The binding was detected by adding alkaline phosphatase substrate nitroblue tetrazolium (Sigma, St. Louis, MO, USA) and 5-bromo-4-chloro-3-indolyl phosphate (Sigma, St. Louis, MO, USA).

### HLA determination

Genomic DNA was extracted from EDTA-treated peripheral blood samples. HLA low/medium resolution typing for HLA-A, HLA-B, HLA-C and HLA-DRB1 and HLA–DQB1 alleles was performed using PCR-sequence specific oligonucleotide (SSO) Luminex LABTYPE®SSO kits (OneLambda, Inc. CA) designed to recognize all the broad specificities based on the sequence databases from IMGT/HLA Database (database version 3.17.0) (http://www.ebi.ac.uk/ipd/imgt/hla/probe.html).


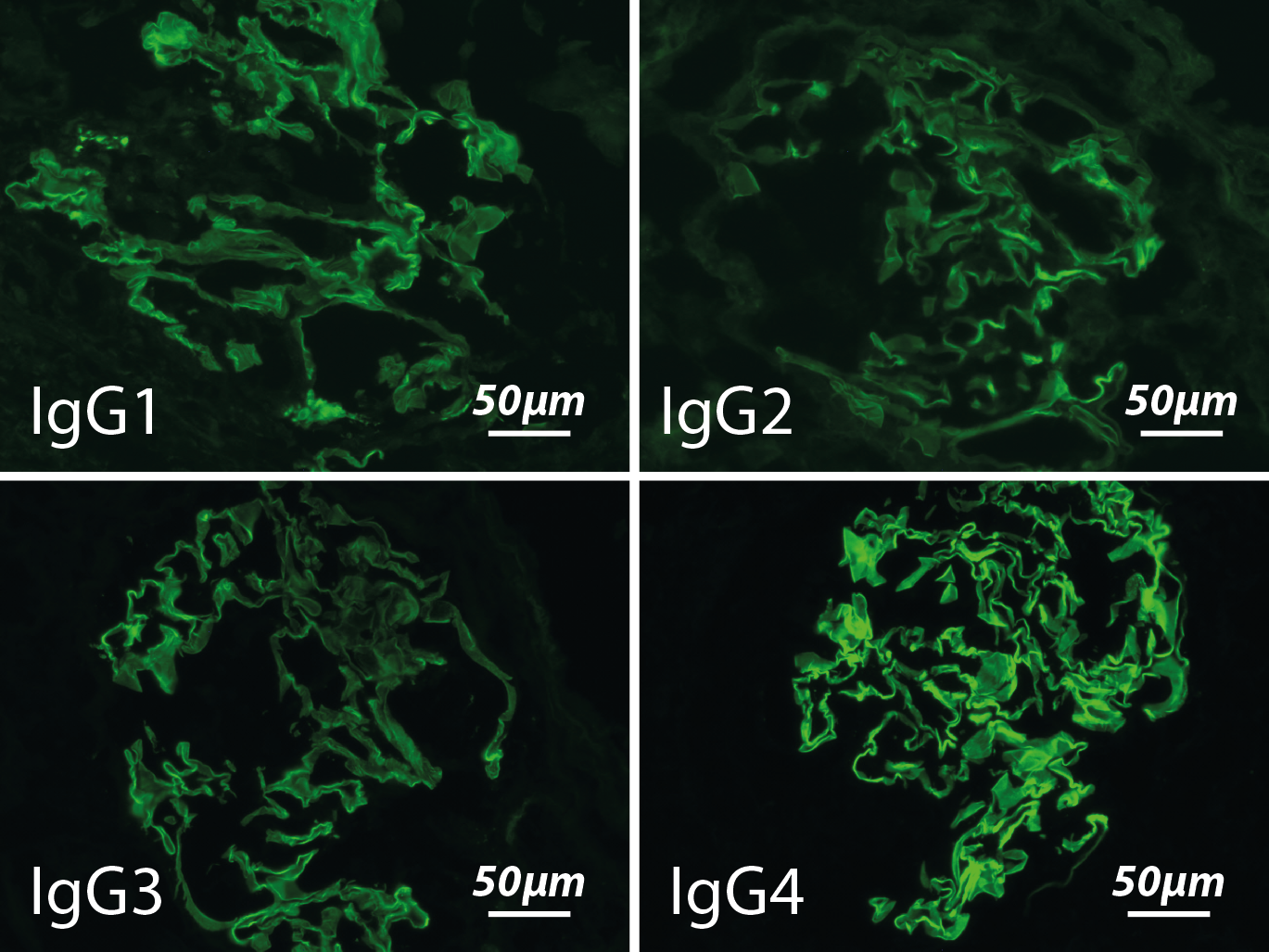


**Figure legend for the immunostaing:**

IgG subclasses immunostaining in a classical, highly proliferative anti-GBM disease. All subclasses are positive.


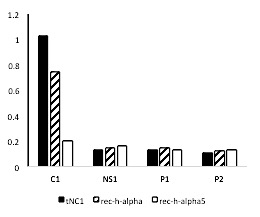


**Figure legend for the ELISA:**

Serum from the patient (P1 and P2) and serum from a heathy control (NS1) showed no reactivity to the different antigens in ELISA. Serum from a known patient with anti-GBM disease (C1) reacted with both bovine typeIV collagen (tNC1) and recombinant alpha3(IV)NC1 domains.


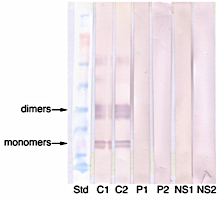


**Figure legend for the western blott:**

Shows a western blott where bovine testis NC1 domains were run on a non-reducing SDS-PAGE gel and transferred to a nitrocellulose membrane and thereafter incubated with sera. C1 and C2 comes from two patients with a known anti-GBM disease and show the characteristic binding to both monomers and dimers of the alpha chains from type IV collagen. Neigher sera from helathy controls (NS1 and NS2) nor the sera from the patient (P1 and P2) showed no binding.
